# First detection of *Amblyomma lepidum* (Dönitz, 1909) in Zimbabwe

**DOI:** 10.1007/s10493-025-01017-7

**Published:** 2025-04-10

**Authors:** Andeliza Smit, Stephen Mandara, Zinathi Dlamkile, Darshana Morar-Leather, Anna-Mari Bosman, Luis Neves

**Affiliations:** 1 Tick Research Group, Department of Veterinary Tropical Diseases, Faculty of Veterinary Science, University of Pretoria, Onderstepoort, South Africa; 2 Department of Animal Production Sciences, Marondera University of Agricultural Sciences and Technology, Marondera, Zimbabwe; 3 Department of Biomedical Sciences, Faculty of Science, Tshwane University of Technology, Arcadia Campus, Pretoria, South Africa; 4 Biotechnology Center, Eduardo Mondlane University, Maputo, Mozambique

**Keywords:** Ticks, Southern Africa, Phylogenetics, 12S rRNA, 16S rRNA

## Abstract

Herewith we present the first detection of *Amblyomma lepidum* (Dönitz, 1909) on cattle in Zimbabwe. Zimbabwe’s smallholder farmers rely heavily on their livestock, mainly cattle and goats. Even though great importance is placed on cattle farming, no systematic surveillance of tick and tick-borne diseases is currently being conducted in the country. Forming part of the *Amblyomma* genus, *A*. *lepidum* is a brightly ornate tick with great vector potential, which could have harmful effects on cattle and cattle farming in Zimbabwe. This report documents the presence of both male and female *A*. *lepidum* ticks that were morphologically identified, and with the use of two ribosomal genes, were molecularly characterised as *A*. *lepidum*. Hypotheses can be made as to how this tick was introduced into Zimbabwe; however, its presence highlights the need for a systematic surveillance programme to track not only new introductions, such as the aforementioned, but the current distribution of this tick population in Zimbabwe.

**Supplementary Information:**

The online version contains supplementary material available at 10.1007/s10493-025-01017-7.

## Introduction

**Fig. 1:**
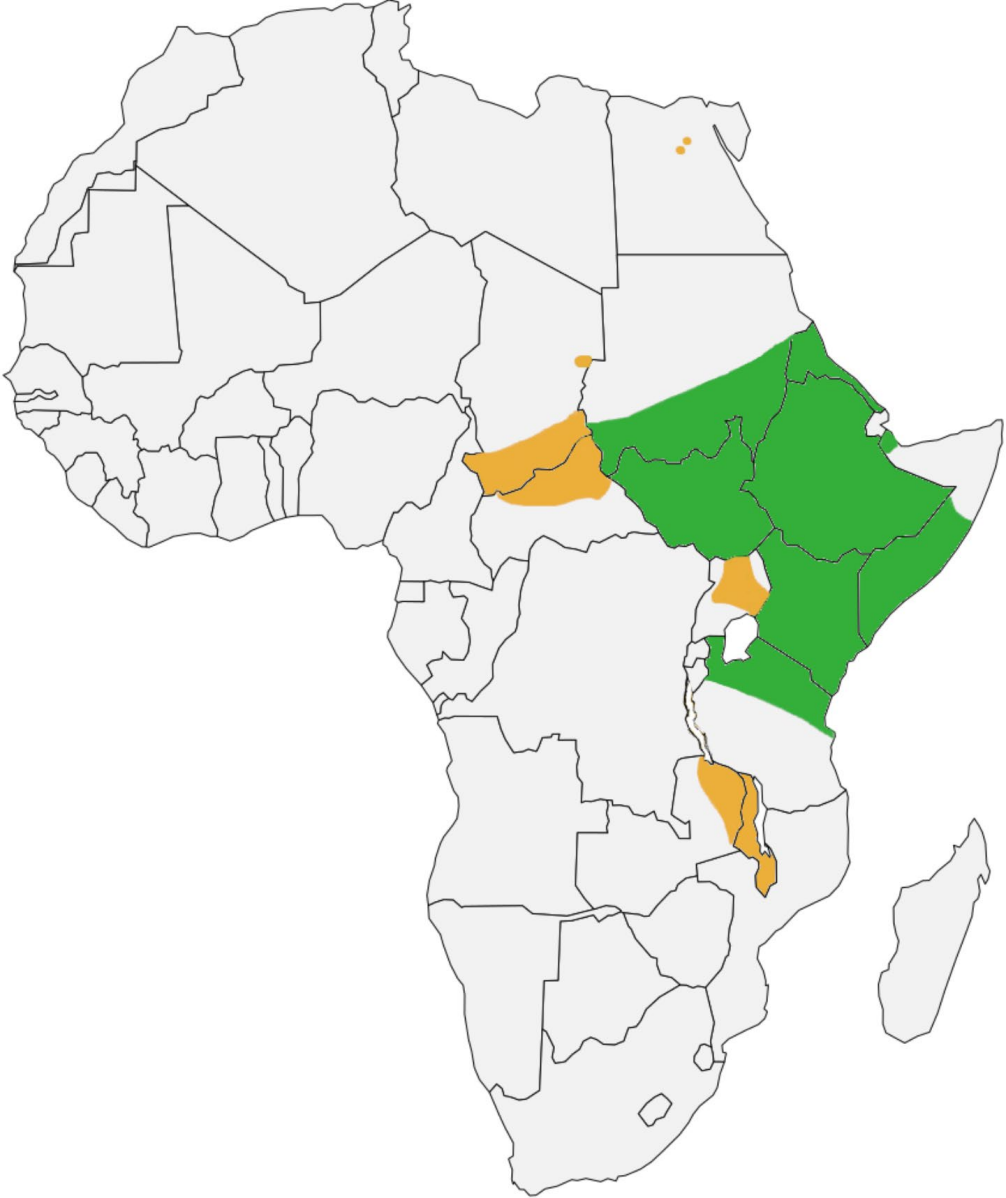
Map of Africa (obtained from https://simplemaps.com/resources/svg-africa) illustrating both the well (green) and less (orange) documented distribution of *Amblyomma lepidum*. Image was constructed with information from Walker and Olwage (1987) and Guglielmone et al. (2023)

*Amblyomma*, one of the largest genera in the Ixodidae, are known for their decorative appearance, aggressive hunting behaviours and vector importance (Walker 1991). To date, 136 *Amblyomma* species have been documented occurring in the Neotropical, Afrotropical, and Australasian faunal regions (Guglielmone et al. 2023). Of the aformentioned 136 species, 24 are known to occur in Africa (Guglielmone et al. 2023). *Amblyomma lepidum* is documented as an East African tick species, occuring from eastern Sudan to northern Tanzania, including Zanzibar (Robinson 1926; Hoogstraal 1956; Walker et al. 2003) (Fig. 1). New distributions have been documented in the Central African Republic (Uilenberg et al. 2013), Zambia (Tandon 1991), Egypt (Hoogstraal 1956; Abouelhassan et al. 2023) and Iran (Piazak 2005), believed to be due to importation of infected cattle or dromedary camels from these east African countries (Abouelhassan et al. 2023, 2024). *Amblyomma lepidum* plays a crucial role in the veterinary field due to the importance in the transmission of several pathogens including *Ehrlichia ruminantium* (Suliman 2011), *Mycobacterium farcinogenes* (Hasabelrasoul et al. 2015) and both *Theileria mutans* and *Theileria velifera* (Musisi 1979). *Amblyomma lepidum* primarily parasitise a large variety of hosts such as cattle, sheep, goats, camels and several wild ungulates (Walker et al. 2003).

*Amblyomma* ticks, including *A*. *lepidum*, pose significant challenges to livestock health and management across various African regions. Tick-borne diseases have a substantial impact on the agricultural sector in developing countries such as Angola, Botswana, Mozambique, Zambia, and Zimbabwe. Often, the farmers lack resources for effective tick control measures and may experience reduced productivity of their animals, decreased market value, and mortality due to tick-borne diseases (Muvhuringi et al. 2022).

Zimbabwe is a landlocked country in southern Africa bordered by South Africa in the south, Mozambique in the east, Botswana in the west, and Zambia in the north. In Zimbabwe, in terms of livelihood, cattle are regarded as the most important livestock, and goats second. Currently, the country boasts a cattle population of an estimated four to five million, of which 89% are located in communal areas belonging to smallholder farmers (Chiremba and Masters 2003; Mavedzenge et al. 2006). The smallholder farmers generally engage in subsistence mixed farming, where cattle are not only an indication of wealth, but are used as a source of protein, milk and manure production, draught power, tillage, transport, and hides (Chiremba and Masters 2003; Mavedzenge et al. 2006). Due to the high importance placed on cattle farming, both parasites and the diseases that they can cause are a significant constraint to communal farmers.

The most commonly reported diseases by these smallholder farmers includes blackleg (*Clostridium chauvoei*), heartwater (*E. ruminantium*), babesiosis, anthrax (*Bacillus anthracis*), and anaplasmosis (Mavedzenge et al. 2006). *Ehrlichia ruminantium* (formerly known as *Cowdria ruminantium* and *Rickettsia ruminantium*) is one of the main causes of the economic loss observed in the cattle industry, alongside East Coast fever, anaplasmosis,

and babesiosis (Jongejan and Uilenberg 2004; Strydom et al. 2023). Heartwater is believed to cause an estimated loss of US$ 5,6 million in 1999 in Zimbabwe (Mukhebi et al. 1999) and US$ 70,4 million in 2022 in South Africa (van den Heever et al. 2022). *Ehrlichia ruminantium* is known to be transmitted by ticks from the genus *Amblyomma*, particularly *Amblyomma hebraeum* and *Amblyomma variegatum*, both of which occur in Zimbabwe (Petney et al. 1987; Walker and Olwage 1987; Peter et al. 1998).

The tick populations in Zimbabwe were previously well documented (Norval 1979; Peter et al. 1998; Mukhebi et al. 1999; Bazarusanga et al. 2007; Estrada-Peña et al. 2008; Hove et al. 2008; Sungirai et al. 2015, 2017), with the most current spatial distribution study on 55,133 ticks in Zimbabwe indicating 14 different species, including *A. hebraeum, A. variegatum, Haemaphysalis elliptica, Hyalomma rufipes, Hyalomma truncatum*,* Rhipicephalus appendiculatus, Rhipicephalus compositus, Rhipicephalus decoloratus, Rhipicephalus evertsi evertsi, Rhipicephalus microplus, Rhipicephalus pravus, Rhipicephalus sanguineus sensu lato, Rhipicephalus simus*, and *Rhipicephalus zambeziensis* (Shekede et al. 2021). Although the historic documentation of tick distribution was well established, currently no regulations are instituted for the systematic surveillance and control of tick-borne diseases in Zimbabwe (Shekede et al. 2021).

This communication serves as the first report of *A. lepidum* in Zimbabwe during a tick surveillance study conducted in 2017.

## Materials and methods

**Fig. 2:**
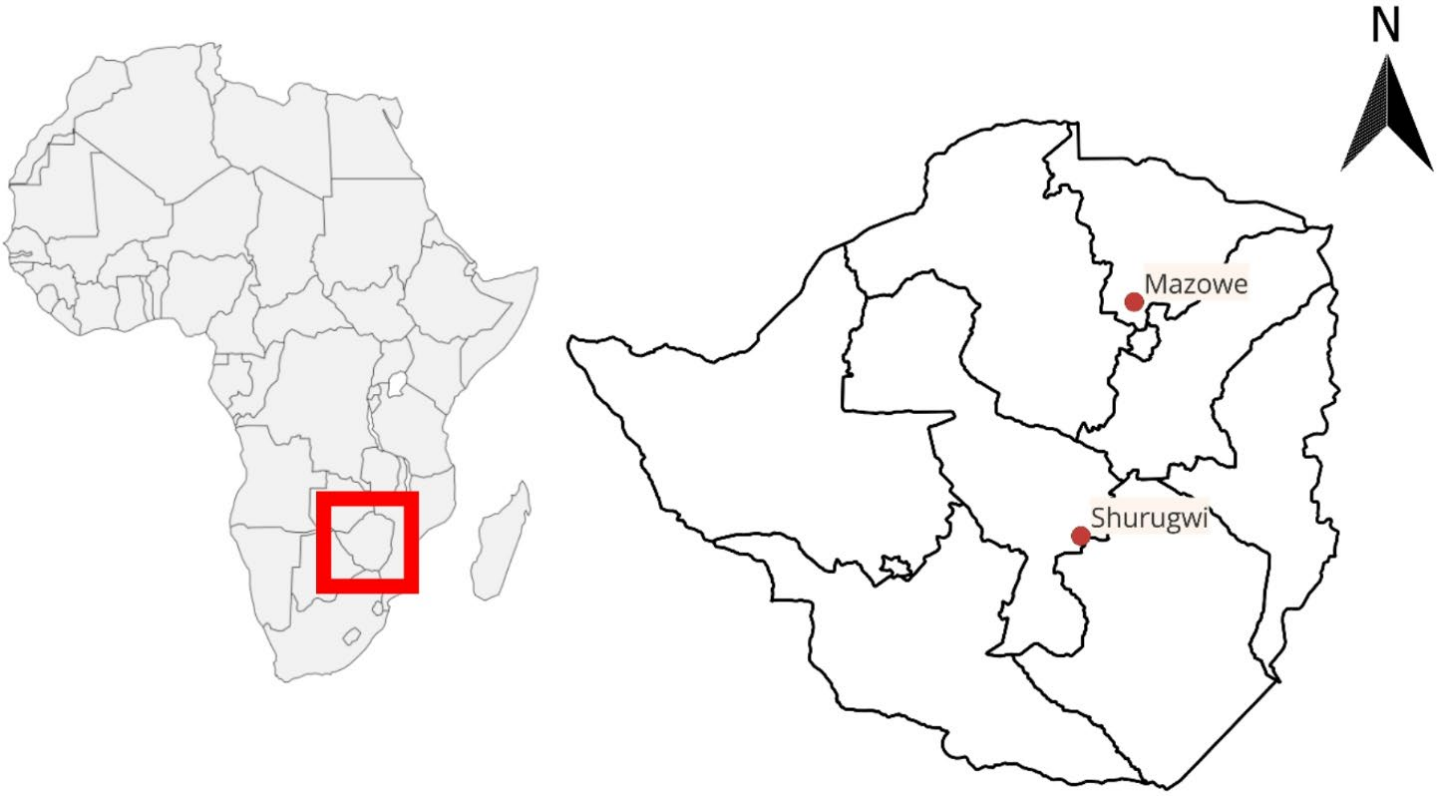
Map of Zimbabwe illustrating the two sampling points of *Amblyomma lepidum*, Mazowe and Shurugwi (Red dots). Figure was created by Ms. Rebecca Ackermann

This study forms part of a previous study, Mandara (2018), in which *Amblyomma* ticks were collected from cattle at multiple locations in Zimbabwe. Sampling was conducted from May to June of 2017 in Shurugwi (19°49’31.9"S, 30°25’27.1"E) and Mazowe (17°27’16.8"S, 30°57’50.3"E), Zimbabwe (Fig. 2). Ticks were collected from cattle, before they entered the diptank, from predilection sites, such as the ears, dewlap, groin, udder, and around the perineum. All collected ticks were preserved in 70% ethanol, ensuring that the ticks from different diptanks and villages were kept separate. Ticks were identified to species level microscopically, with the use of identification keys obtained from Walker et al. (2003) and Horak et al. (2018). Three *Amblyomma* sp. were documented of which only samples identified as *A. lepidum* were subjected to further molecular analysis to confirm identification for this manuscript. Compounded photograph of *A*. *lepidum* ticks were taken using the Nikon SMZ25 stereomicroscope and NIS-Elements (version 5.21) program. Image backgrounds were removed using the gnu image manipulation program (GIMP) (Solomon 2009) version 2.10.38.

DNA was extracted using the Chelex 100 resin (Bio-Rad, USA) method as described by Smit et al. (2023) from one/two legs per tick. For molecular characterisation, DNA was amplified using two molecular markers, 12S rRNA (Beati and Keirans 2001) and 16S rRNA (Black and Piesman 1994) (Table 1). PCR cycling conditions where used as previously described by Smit et al. (2024). The general PCR cycling conditions for each marker comprised an initial denaturation at 98 °C for 10 s, followed by 10 cycles of denaturation at 98 °C for 1 s, annealing at x °C for 5 s and extension at 72 °C for 15 s. This was followed by 30 cycles of amplification with denaturation at 98 °C for 1 s, annealing at y °C for 5 s and extension at 72 °C for 15 s. Final extension was performed at 72 °C for 15 s.

**Table 1:** 12S rRNA and 16S rRNA primer information including the target region, the primer name, primer length, primer sequence, the expected product size, reagent volumes, and annealing temperature

Target gene	Primer Name	Primer sequence (5’to3’)	Expected fragment size	Volumes (µL)	Annealing temperature (°C)
				(H_2_O, Polymerase*, each Primer, DNA)	(x °C, y °C)
*12S*	T1B	AAA CTA GGA TTA GAT ACC CT	380–400 bp	8, 10, 0.5^a^, 1	60 °C, 49 °C
	T2A	AAT GAG AGC GAC GGG CGA TGT			
*16 S*	16 S + 1	CTG CTC AAT GAT TTT TTA AAT TGC TGT GG	320–460 bp	8, 10, 0.5^a^, 1	48 °C, 54 °C
	16 S-2	TTA CGC TGT TAT CCC TAG AG			

* Phusion Flash High Fidelity Master Mix (Thermo Fisher Scientific., USA), Final concentration 1X

^a^Primer final concentration 0.5 µM

Amplicons were mixed with DNA gel loading dye (X6) (Thermo Fisher Scientific., USA) and separated alongside a 100 bp DNA ladder (Thermo Fisher Scientific., USA) on a 1.5% agarose gel and visualised using the Bio-Rad gel documentation system with an assisting visualisation programme. Samples that produced a clear single band at the expected size were considered positive. All positive samples were sent to the Central Analytical Facilities, Stellenbosch University, Stellenbosch, South Africa for Sanger sequencing.

*Amblyomma lepidum* sequences for 12S rRNA and 16S rRNA were viewed and edited, and contigs were constructed using CLC main workbench version 24.0.2 (developed by CLC Bio, http://www.clcbio.com). Reference sequences were obtained from GenBank (https://www.ncbi.nlm.nih.gov/genbank/) (Sayers et al. 2025) and from the tick sequence reference data base (Department of Veterinary Tropical Diseases, Faculty of Veterinary Science, University of Pretoria, Onderstepoort) (unpublished data) (Online Resource 1). Assembled matrices for each gene were aligned with the use of the online version of MAFFT version 7 (developed by http://mafft.cbrc.jp/alignment/server/index.html) (Katoh and Standley 2013) with default parameters. *Dermacentor rhinocerinus* was selected as the outgroup. The aligned matrices were manually viewed, edited and truncated using CLC main workbench version 24.0.2. Evolutionary model estimations were conducted by using ModelFinder (Kalyaanamoorthy et al. 2017).The 12S rRNA topology was constructed using the TPM3 + F + G4 model, while the 16S rRNA topology was constructed using the TVM + F + G4 model. Maximum likelihood (ML) analysis was conducted on IQ-Tree webserver (Trifinopoulos et al. 2016) with ultrafast bootstrap calculations (Hoang et al. 2018). Resulting topologies were viewed and edited using iTOL (Letunic and Bork 2006,2024).

## Results

**Fig. 3:**
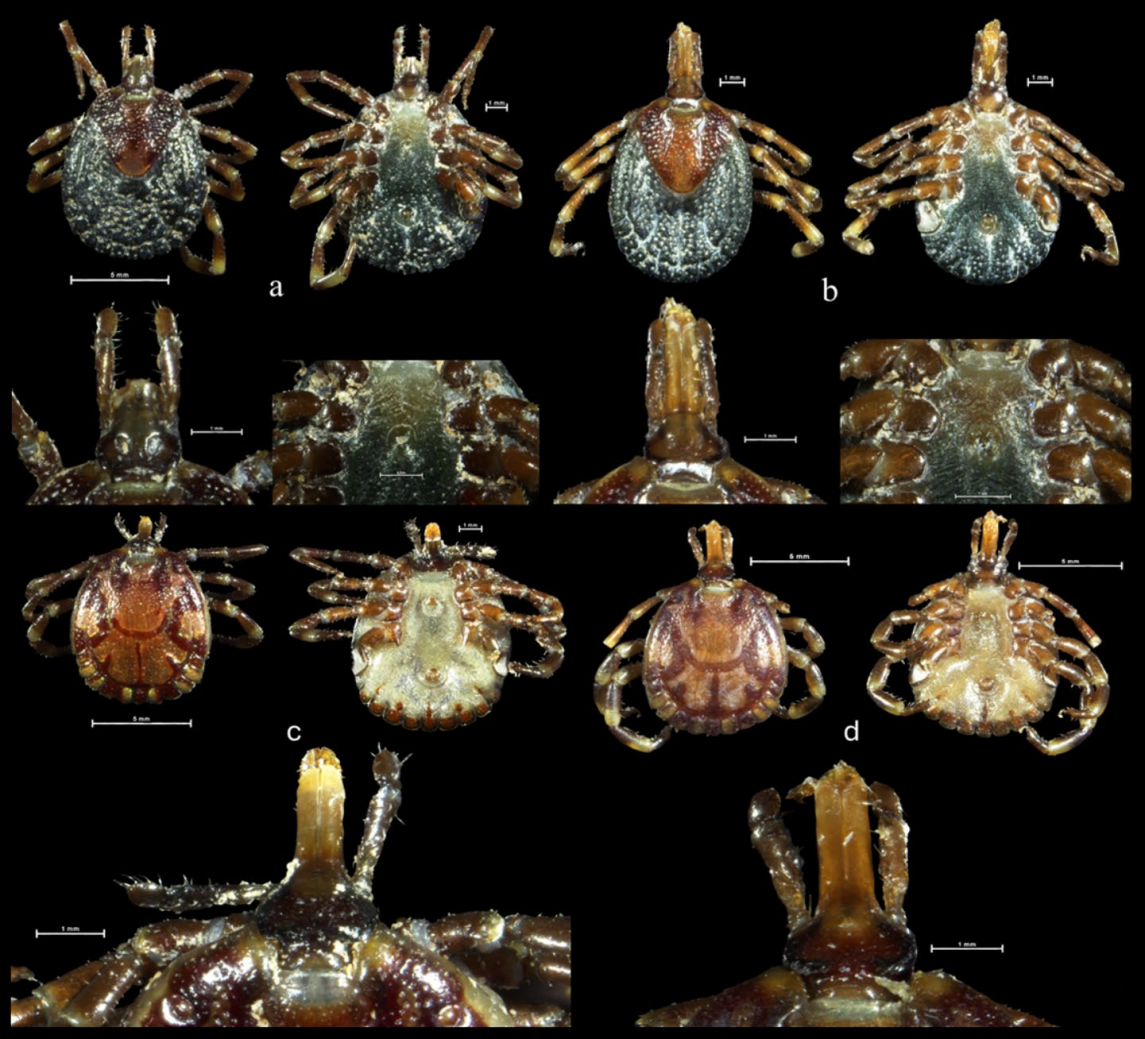
Composite photographs of *Amblyomma lepidum* females (Fig. 3a– sample T2F2 and Fig. 3b– sample T34F2) dorsal, ventral, capitulum and genital apperture views. composite photographs of *A. lepidum* males (Fig. 3c– sample T2M3 and Fig. 3d– sample T34M1) dorsal, ventral and capitulum views. Both 3a and 3c were collected from cattle in Shurugwi, while 3b and 3d were collected in Mazowe, Zimbabwe

In the previous study, Mandara (2018) collected a total of 194 *Amblyomma* ticks which were morphologically identified to belong to three different species, including *A*. *hebraeum* (85.6%), *A*. *lepidum* (9.8%) and *A*. *variegatum* (4.6%). This manuscript will only focus on the *A*. *lepidum* that was collected in the aforementioned study. From 28 *A*. *lepidum* ticks that were collected, 11 were female and 17 were male. In Shurugwi three females and 12 males were collected, while in Mazowe eight females and five males were collected. A clear morphological differentiation between *A. lepidum*,* A*. *hebraeum* and *A*. *variegatum* is the colouration of the festoons. *Amblyomma lepidum* has partially enamelled festoons, whereas nine of the 11 festoons of *A*. *hebraeum* has complete enamelling and *A*. *variegatum* have festoons with no enamelling (Voltzit and Keirans 2003; Walker et al. 2003) (Fig. 3a). The other main characteristic of male *A*. *lepidum* ticks are documented in Fig. 3a and includes distinctively convex eyes, long mouthparts, bold enamel ornamentation with spots on lateral sides, adanal plates are absent, legs are dark in colouration with pale bands and coxae I and IV have a pair of spurs. Female *A*. *lepidum* ticks (Fig. 3b) resemble those of *A*. *variegatum*, however, a larger, more elongated enammeled patch is present at the posterior end of the scutum (Voltzit and Keirans 2003; Walker et al. 2003).

The BLAST analysis of *A*. *lepidum* mitochondrial fragments of the 12S rRNA gene confirmed the morphological identification, revealing best hits (100% nucleotide identity with a 94% query cover) with *A*. *lepidum* sequences from Kenya (GenBank accession no. OQ565136, OQ565143). While the BLAST analysis of *A*. *lepidum* mitochondrial fragments of 16S rRNA gene confirmed the morphological identification, revealing best hits (99.36% nucleotide identity with a 95% query cover) with *A*. *lepidum* sequences from Kenya and Somalia (GenBank accession no. OQ566203, ON532095, respectively).

**Fig. 4:**
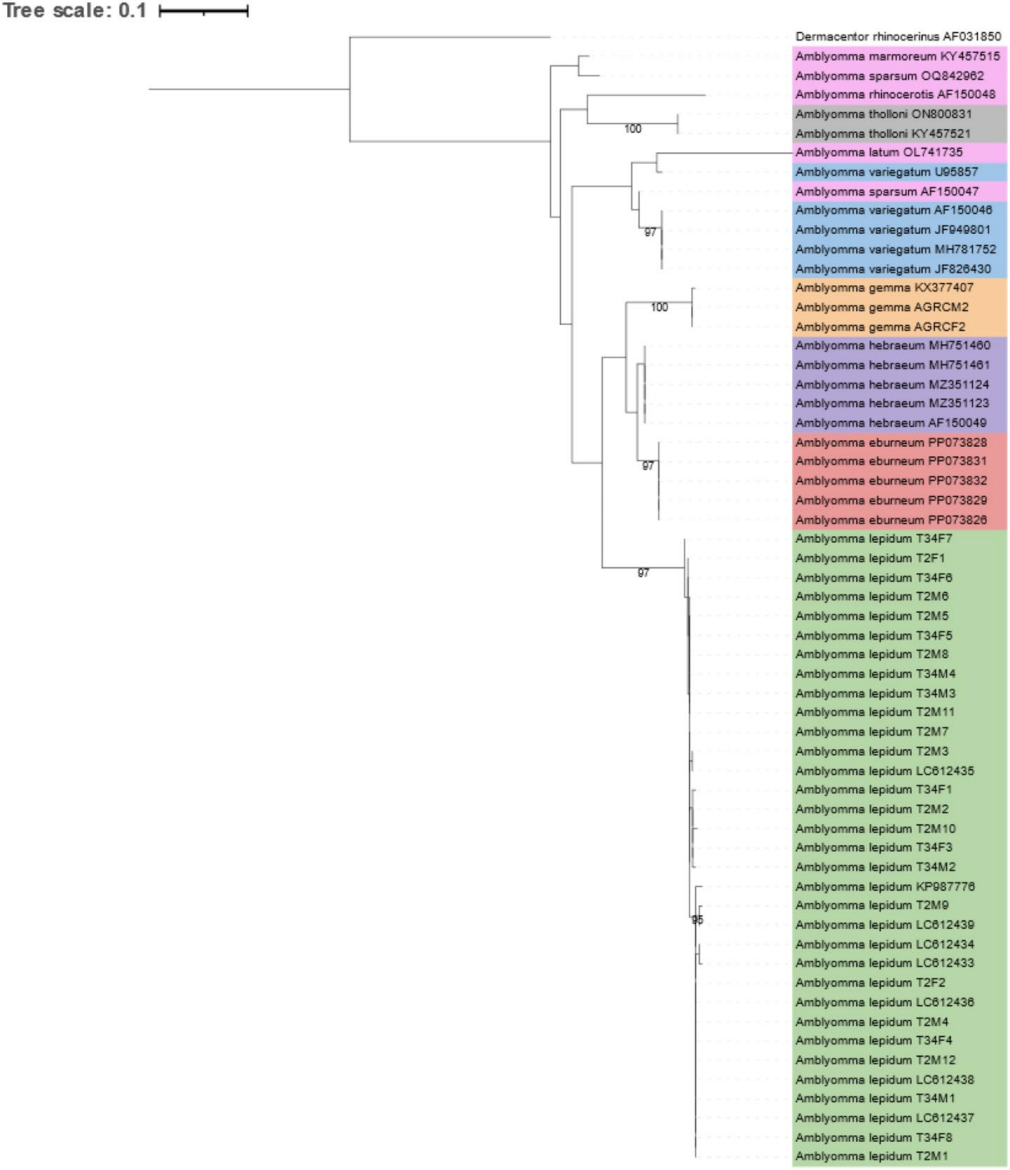
Phylogenetic inference based on the 12S rRNA gene (TPM3 + F + G4 model). Colour corresponds to species: *Amblyomma eburneum* (red), *Amblyomma gemma* (yellow), *Amblyomma hebraeum* (purple), *Amblyomma lepidum* (green), *Amblyomma tholloni* (grey), *Amblyomma vairgatum* (blue) and other *Amblyomma* sp (pink). Sample names consist of species name and sample number or accession number. Note in sample numbers, M indicates Males, F indicates Females and RC indicates Reference Collection ticks

**Fig. 5:**
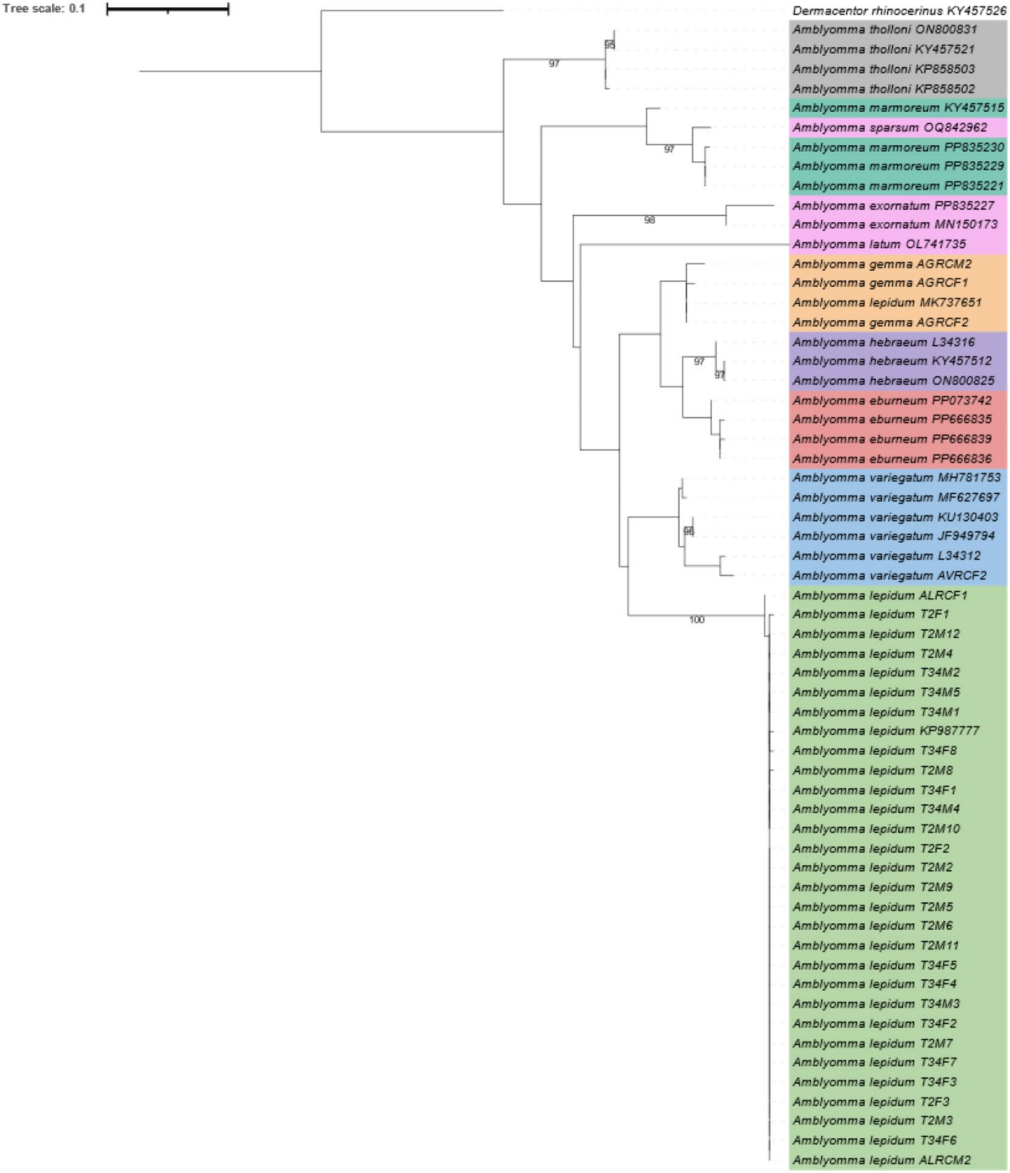
Phylogenetic inference based on the 16S rRNA gene (TVM + F + G4 model). Colour corresponds to species: *Amblyomma eburneum* (red), *Amblyomma gemma* (yellow), *Amblyomma hebraeum* (purple), *Amblyomma lepidum* (green), *Amblyomma marmoreum* (teal), *Amblyomma tholloni* (grey), *Amblyomma vairgatum* (blue) and other *Amblyomma* sp (pink). Sample names consist of species name and sample number or accession number.Note in sample numbers, M indicates Males, F indicates Females and RC indicates Reference Collection ticks

Analysis of the 12S rRNA topology depicted clear clustering of the sample sequences from this study and *A*. *lepidum* reference sequences (those from GenBank) (Bootstrap support of 97) (Fig. 4). The 12S rRNA topology depicts clear separation of *A*. *lepidum* from all other *Amblyomma* species, forming well defined species clades for *A*. *lepidum, A*. *hebraeum, A eburneum* and *A*. *gemma*. Majority of the *A*. *variegatum* clusters within its own well supported clade (Bootstrap of 97), except for reference sequences U95857 which clusteres with *Amblyomma latum*. Other *Amblyomma* sp. sequences have insufficient representatives to make conclusive remarks. The 16S rRNA topology depicts a clear clustering of *A*. *lepidum* samples obtained in this study with one reference sequence obtained from GenBank (bootstap value of 100) (Fig. 5). One *A*. *lepidum* sequence (MK737651) clusters within the *A*. *gemma* clade. Majority of the species clustered in well defined clades, while *A*. *sparsum* was observed clustering within the *A. marmoreum* clade.

## Discussion

Herewith, we report the first identification and geographic distribution of *A. lepidum* in Zimbabwe. The identification of the collected ticks was confirmed by morphology (Fig. 1) and with molecular analyses of 12S rRNA (Fig. 2) and 16S rRNA (Fig. 3).

Morphological identification alone has been noted to lead to misidentifications, especially between species with similar features. Both *A*. *gemma* and *A*. *lepidum* are very similar in appearance and this can lead to the misidentification when trying to identify between these two species (Walker et al. 2003). Since no record of the occurrence of either of these species was made previously in Zimbabwe, confirmation using molecular phylogenies was required. Phylogenetic analysis of both mitochondrial genes depicted a single clade, with sequences from this study clustering with sequences of *A*. *lepidum* from North-Eastern Africa (Kenya, Sudan and Uganda), possibly indicating the origin of the introduction. During the 16S rRNA phylogenetic construction, one of the GenBank reference sequences (MK737651) clustered within the *A*. *gemma* clade. This could be due to the extreme morphological similarity, as mentioned earlier, between the two species and might be a misidentification by the original submission (Direct submission).

As mentioned previously, *A. lepidum* is documented as an East African species that prefers arid and savannah regions (Petney et al. 1987; Walker and Olwage 1987) (Fig. 1). The distribution of *A. lepidum* has been recorded to span from central and eastern Sudan, through Ethiopia, southern Somalia, eastern Uganda, Kenya, and the northern part of central Tanzania (Petney et al. 1987). Rainfall has been noted to be a keystone factor in the distribution range of *A*. *lepidum*; where 250 to 750 mm per annum (p.a) is considered to be the ideal range, but can tolerate up to 1,250 mm p.a (Petney et al. 1987; Walker and Olwage 1987; Abouelhassan et al. 2024). Samples were collected in Shurugwi (in the south) and Mazowe (central) in Zimbabwe. Shurugwi is documented to receive between 600 and 700 mm rainfall p.a, while Mazowe is documented to receive 800 to 900 mm rainfall p.a (Mazvimavi 2010). Given the abundance of cattle and the climate in Zimbabwe, it is an ideal location for *A*. *lepidum*.

Several hypotheses have been linked to new introductions and an increase in the geographical distribution of ticks. The most preferred explanation being climate change, however, unrestricted animal movement has been documented to be a main source of new introductions ticks and their expanded geographical distributions (Nyangiwe et al. 2018). With the new occurrence of *A*. *lepidum* in Zimbabwe, we pose two main explanations. The first, and least likely possibility, could be that the immature stages of the tick may have detached from their hosts, possibly migratory birds from North-Eastern Africa. With the favorable weather along with an abundance of cattle, these ticks could establish themselves easily in the country. The second, and most likely scenario, includes the transportation of infested ungulates, mainly cattle, from North-Eastern Africa to Zimbabwe, where *A*. *lepidum* once again establishes a viable population due to favorable weather conditions and an abundance of hosts. Due to the inadequate surveillance and control of ticks and tick-borne diseases in Zimbabwe, as indicated by Shekede et al. (2021), the introduction of an infested cattle is most probably the source of the introduction and geographical spread of *A. lepidum* (Abouelhassan et al. 2024) in Zimbabwe.

Vector competence studies of *A*. *lepidum* are few. However, both *E. ruminantium* and *Anaplasma bovis* species have been detected in this tick (Walker and Olwage 1987; Teshale et al. 2015). Both *Ehrlichia* and *Anaplasma* can be detrimental to the cattle industry in Zimbabwe. Thus, we propose that a surveillance program be implemented to determine and track the current tick populations and their geographical spread.

To conclude, this study documents the first report on the occurrence of *A*. *lepidum* in Zimbabwe. Both males and females of the species were collected, indicating potentially well-established populations at both collection points. Identification was confirmed with both morphology and molecular analyses (12S rRNA and 16S rRNA). This reporting highlights the need for more comprehensive and thorough tick surveillance campaigns in Zimbabwe.

## Electronic supplementary material

Below is the link to the electronic supplementary material.

Supplementary Material 1

## Data Availability

Sequence data that support the findings of this study have been deposited in the GenBank repository, accession numbers can be found within the manuscript and supplementary information file.
